# Do acute postoperative seizures predict epilepsy surgery outcome? a scoping review

**DOI:** 10.1007/s00701-025-06486-8

**Published:** 2025-03-13

**Authors:** Sebastiaan E. A. van Maanen, Maeike J. M. Zijlmans, Pieter van Eijsden, Sandra M. A. van der Salm

**Affiliations:** Department of Neurology and Neurosurgery, Utrecht Brain Center, University Medical Center Utrecht, 3584CX Utrecht, Netherlands

**Keywords:** Epilepsy, Neurosurgery, Postoperative period, Review, Seizure disorder

## Abstract

**Background:**

Acute postoperative seizures (APOS) are common phenomena following resective epilepsy surgery and can be categorized as running-down (RDS) or running-up seizures (RUS). This differentiation is made retrospectively, considering their classification is based on seizure recurrence. However, early differentiation of RDS from RUS may prevent unnecessary escalation of anti-seizure medication or reoperation. This review provides an overview of the available literature on variables influencing the evolution to RDS/RUS in patients exhibiting acute or early postoperative seizures.

**Methods:**

A database search was performed addressing studies related to the running-down phenomenon and postoperative seizures in PubMed and Embase. Eligibility required a clear definition of acute or early postoperative seizures. Studies concerning any type of epilepsy surgery or pathology were accepted, excluding those related to high-grade malignancies.

**Results:**

The search yielded a total of *n* = 1,690 records. We included *n* = 21 studies with a total of *n* = 1,496 patients, which examined variables associated with long-term seizure outcome. Interictal epileptiform discharge presence/laterality, epileptogenic zone size, APOS frequency, and history of generalized tonic–clonic seizures, head trauma, or encephalitis were associated with seizure outcome. Ictal expression and timing of seizure recurrence appeared less relevant. However, these associations are uncertain due to conflicting results between studies, likely due to small sample sizes, a limited reporting of secondary variables, and heterogeneity in study population and methodology.

**Conclusions:**

The variability in clinical outcome following APOS highlights the need for a refined classification of postoperative seizures. Future research should focus on constructing and validating a multifactorial model integrating EEG-derived variables, APOS frequency, and medical history to more accurately predict long-term seizure outcome following resective epilepsy surgery.

**Supplementary Information:**

The online version contains supplementary material available at 10.1007/s00701-025-06486-8.

## Introduction

Resective surgery is typically the most effective and preferred treatment for epilepsy; however, many patients do not achieve complete seizure remission postoperatively. In studies performed between 1990–2020, the pooled proportion of patients becoming seizure-free following temporal lobe surgery was 61% for children and 56% for adults [1]. 13% of both adult and pediatric patients were classified as Engel IB/IC/ID, [1] indicating non-disabling seizures or seizures occurring only in the early postoperative phase [9]. Acute postoperative seizures (APOS) are defined as ictal events occurring within the first week postoperatively [2] and are strongly associated with long-term seizure recurrence [12]. However, up to 40% of the patients exhibiting APOS demonstrate a favorable clinical outcome in the long term, indicating heterogeneous disease courses [12]. This running-down of seizures envelops the presentation of postoperative seizures gradually decreasing in frequency until, ultimately, remission is achieved [32]. These seizures show a considerable diversity in their timing of onset, though they are known to fully remit within 2 years [13, 28, 33, 34]. Differentiating running-down seizures (RDS) from their contrasting running-up seizures (RUS) can only be done retrospectively, as their classification depends on eventual seizure recurrence. Following resective surgery, this clinical paradox creates caregiver uncertainty, as it remains unclear whether postoperative seizures are self-limiting or require intervention. Recognizing RDS at an early stage is therefore essential for clinical decision-making, as it prevents unnecessary escalation of anti-seizure medication or reoperation. However, it has not yet been clarified which factors determine either course of disease or which predictors can be applied in a clinical setting. This is partly attributable to the unresolved pathophysiology of RDS. The most accepted theory was postulated by Rasmussen, stating that RDS are caused by epileptogenic tissue surrounding the surgical resection site [32]. This tissue, initially affected through secondary epileptogenesis, would not be capable of autonomously generating seizures indefinitely after removal of the primary epileptogenic zone (EZ). The adjacent tissue would then recover from its previously induced synaptic dysfunction, reflected by a gradually decreasing seizure frequency [32]. RUS, on the other hand, may be a result of independent epileptogenic tissue persisting due to either incomplete resection or the maturation of novel foci.

Identifying predictors of RDS/RUS will alleviate the uncertainty of whether seizures will eventually cease to occur, benefit clinical counseling, and facilitate therapeutic interventions in a more timely manner. Hence, distinguishing RDS from RUS may reduce treatment burden as well as improve patient outcome. This review aims to provide an overview of the available literature on variables determinative for the onset of either RDS or RUS in patients exhibiting acute or early postoperative seizures.

## Methods

The review was registered in PROSPERO under registration number 347140. However, it was automatically rejected as it did not focus on COVID-19 registrations during the 2020 pandemic. Given this, another registration was not submitted. The registration states the review would include a meta-analysis of the risk of developing seizures following APOS. This was later amended after it became clear that a meta-analysis on this topic had already been conducted by Giridharan et al., which showed patients without APOS are 4.2 times more likely to be seizure-free after ≥ 1 year [12]. A protocol was not prepared for this review. The current review adheres to the PRISMA 2020 guidelines as far as applicable, considering their intended use is for systematic, rather than scoping reviews.

A database search was performed on 10/28/2024 addressing studies related to the running-down phenomenon and postoperative seizures in PubMed and Embase (Online Resource). The keywords in the search string were “epilepsy”, “seizures”, “epilepsy surgery”, “running down phenomenon”, “running down seizures”, “postoperative seizures”, including their synonyms. Different types of epilepsy-related surgical procedures such as “resection”, “topectomy”, “lobectomy”, “transection”, “amygdalohippocampectomy”, and “lesionectomy” were included.

Literature selection was conducted manually by a single reviewer. First, titles and abstracts were screened to determine whether studies concerned early postoperative seizures. Subsequently, in- and exclusion criteria were applied. Studies were included if their study population consisted of surgically treated patients with epilepsy, and quantified the association between seizure characteristics and RDS/RUS or seizure outcome. Studies were required to clearly define early postoperative seizures/APOS. Studies regarding any type of epilepsy surgery or pathology were eligible, except for those concerning high-grade malignancies. Reports were excluded if they concerned a review or meta-analysis, a case report, a non-human study, or a non-clinical study, and if they were not written in English. Articles were also excluded if only their abstract was published, and this did not contain sufficient information on study methodology and outcomes to assess eligibility.

## Results

**Fig. 1:**
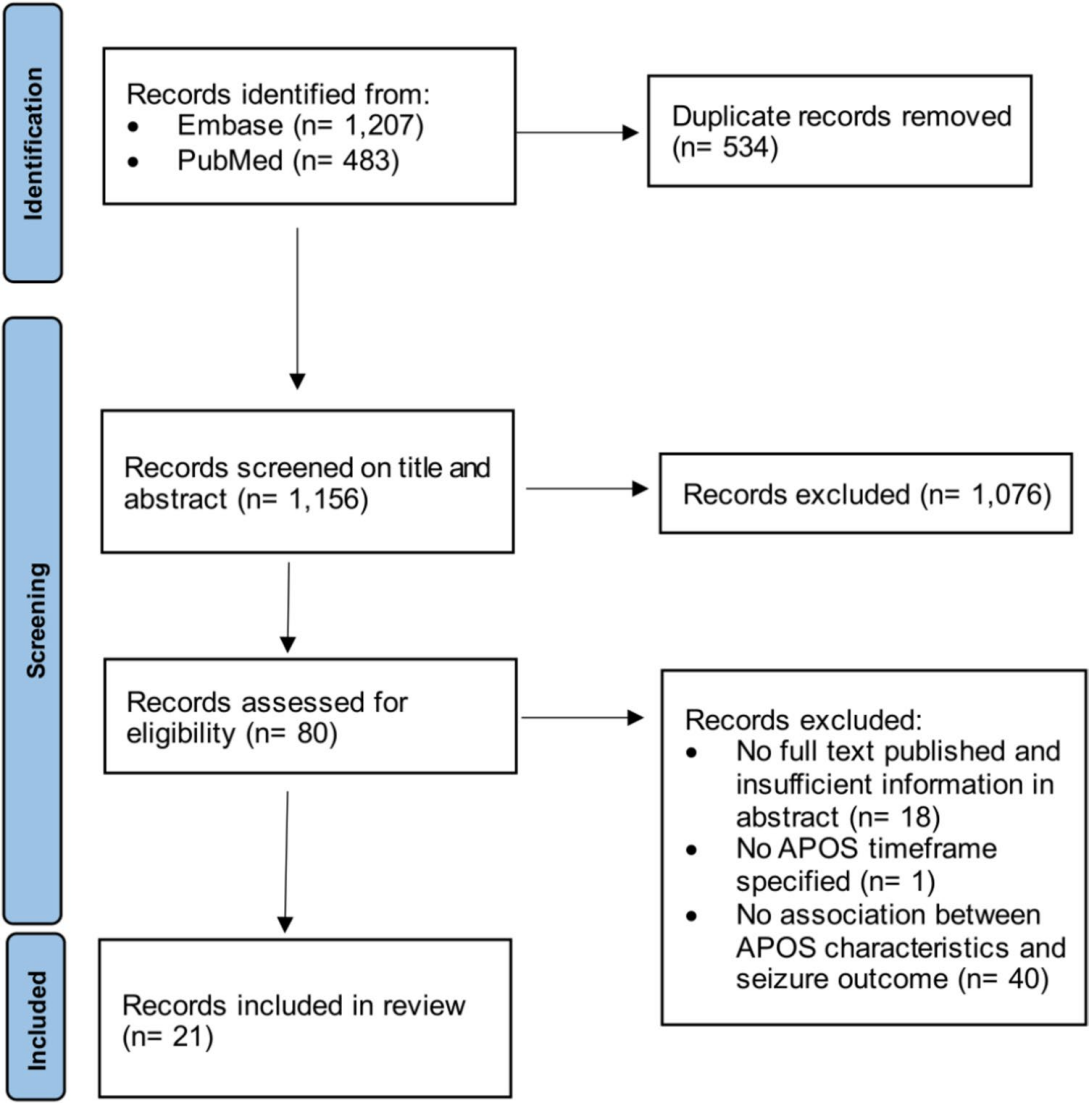
PRISMA flow chart illustrating literature selection process. APOS: acute postoperative seizures

Applying the search string in Embase and PubMed yielded *n* = 1,690 records (Fig. 1). Following duplicate removal, *n* = 1,156 records were subjected to eligibility assessment. After screening title and abstract for relevance *n* = 1,076 reports were excluded. The in- and exclusion criteria were applied to the remaining *n* = 80 articles. As a result, *n* = 59 articles were excluded, of which *n* = 18 had no full text published and supplied insufficient information in the abstract, *n* = 1 had not specified the APOS timeframe, and *n* = 40 had not analyzed an association between APOS characteristics and seizure outcome. A total of *n* = 21 studies were included in this review with a total of *n* = 1,496 patients. Characteristics and key findings of the included studies are presented in Tables 1 and 2.

**Table 1:** Summary of characteristics of included studies (*n* = 21)

Total included studies (*n* =)^b^ Median sample size (IQR)	21 45 (37)
Total patients of interest (*n* =*)*	1,496
Total patients of interest with favorable outcome (*n* =*)* Pooled proportion patients with favorable outcome	548 0.37 (95% CI 0.34–0.39)
Included epilepsy pathologies (number of studies)	Malformation of cortical development (13) • Focal cortical dysplasia (7)^c^ • Microdysgenesis (2) • Tuberous sclerosis (1) • Hemimegaloencephaly (1) Mesial temporal sclerosis/hippocampal sclerosis/gliosis (12) Malignancy (12) Vascular malformation (8) Trauma (3) Ischemia (3) Normal (8)
Included surgical procedures (number of studies)	Temporal lobectomy/cortical resection (14) Extratemporal cortical resection (5) Lesionectomy (4) Amygdalohippocampectomy (4) Hemispherectomy (4) Key-hole resection (1)
Included variable of interest (number of studies)	APOS semiology (8) Focus of epilepsy (3) EEG (6) MRI (4) Timing of seizure recurrence (9) Seizure frequency (6) Patient characteristics (5)

^a^ *APOS* Acute postoperative seizures

^b^ Mean length of follow-up is not presented in this table, as this variable could not be pooled

^c^ Except for the study by Jin et al. [21], no distinction was made between subtypes of focal cortical dysplasia

**Table 2:** Study characteristics and key findings of included studies (*n* = 21)

Authors, year of publication, affiliated institution	Population	Epilepsy pathology (*n* =)	Surgical procedure (*n* =)	Mean follow-up in months (± SD/range)	Patients of interest (*n* =)^b^	Patients of interest with favorable outcome (*n* =)^c^	Key findings
[14], Cincinnati Children’s Hospital Medical Centre (USA)	Patients aged < 21 years undergoing resective surgery for epilepsy	Normal, focal cortical dysplasia (25), tuberous sclerosis, ischemia, trauma, encephalitis	Hemispherectomy (9), temporal (7), extratemporal (22), multilobar (12) surgery	26 (± 11), minimum of 12	50 (APOS defined as seizures < 30 days postoperatively)	13	1. Postoperative seizure semiology similar to preoperative presentation was a predictor of poor outcome (*p* < 0.001) 2. No significant difference in outcome was found between patients presenting with APOS within < 7 days vs. > 7 days (*p* = 0.104)
[21], Sanbo Brain Hospital of Capital Medical University (China)	Patients who had intractable epilepsy and underwent resective surgery	Focal cortical dysplasia type I-III (21)	N/A	34.6 (± 15.4), minimum of 12	21	9	Habitual APOS were strongly associated with seizure recurrence (RR 3.55, 95% CI 1.59–7.94)
[25], Mayo Clinic (USA)	Patients with intractable temporal lobe epilepsy who underwent resective surgery	N/A	Anterior temporal lobectomy and amygdalohippocampectomy (32)	37.4 (range 12–94)	32	20	1. Patients with APOS identical to preoperative seizures were less likely to have a favorable long-term outcome (RR 0.19, 95% CI 0.019–1.89) 2. No significant difference in outcome was found between those with and without provoked seizures (RR 2.17, 95% CI 0.75–6.28) 3. No significant difference in outcome was found between patients with APOS within < 24 h vs. > 24 h (RR 0.81, 95% CI 0.32–2.02)
[37], Mayo Clinic (USA)	Patients with immediate postoperative seizures following anterior temporal lobectomy	Astrocytoma (4), ganglioglioma (1), arteriovenous malformation (3), other etiologies unknown	Anterior temporal lobectomy (37)	33.7 (range 12–68)	37	20	1. Patients with early postoperative generalized seizures had a higher rate of seizure freedom during follow-up than those with focal seizures with impaired awareness (*p* = 0.11) 2. Young age at seizure onset was associated with seizure freedom (*p* < 0.05) 3. Age at operation and sex were no good predictors of outcome (*p* > 0.05)
[26], Cleveland Clinic Association (USA)	Patients < 18 years who underwent extratemporal epilepsy surgery for medically intractable epilepsy	Cortical dysplasia (22), non-cortical dysplasia (12)	Extratemporal cortical resection (26), hemispherectomy (8)	12	34	7	1. No significant difference in outcome was found between patients with habitual seizures vs. non-habitual seizures (*p* = 0.47) 2. APOS occurring < 24 h showed a more favorable seizure outcome as opposed to > 24 h (*p* = 0.06) 3. No significant difference in outcome was found between patients with single vs. multiple postoperative seizures (*p* = 0.92)
[27], Austin Health (Australia)	Patients who underwent anterior temporal lobectomy	Vascular malformation, astrocytoma, oligodendroglioma, ganglioglioma, neuroepithelial tumor, cyst, hippocampal sclerosis, microdysgenesis, minor global atrophy, distant lesions	Anterior temporal lobectomy (69)	12	69 (APOS defined as seizures < 28 days postoperatively)	16	1. No significant difference in outcome was found between patients with generalized seizures vs. focal seizures with impaired awareness, with or without secondary generalization (RR 1.29, 95% CI 0.6–2.7) 2. Seizure precipitants trended towards risk reduction of later seizure recurrence (RR 0.61, 95% CI 0.34–1.21) 3. No significant difference in outcome was found between patients with APOS onset < 24 h vs. between 2–14 days (RR 1.6, 95% CI 0.76–3.43) 4. No significant difference in outcome was found between patients with APOS onset < 24 h vs. > 14 days (RR 1.0, 95% CI 0.42–2.44). The RR trends to be more favorable compared to seizures occurring between 2–14 days 5. No significant difference in outcome was found between patients with single vs. multiple seizures (RR 1.43, 95% CI 0.8–2.5)
[31], Mayo Clinic (USA)	Patients < 18 years who underwent surgery for the relief of medically intractable epilepsy	N/A	Temporal lobectomy, extratemporal resection, hemispherectomy	> 12	37	17	APOS presentation, timing of seizure recurrence, and postoperative seizure frequency were not reliable predictors of seizure outcome (no effect measure given)
[11], Northern California Comprehensive Epilepsy Center (USA)	Patients who underwent temporal lobectomy for medically refractory epilepsy	Low grade glioma, vascular malformation, mesial temporal sclerosis, normal	Temporal lobectomy	29 (± 11)	27	12	1. Seizure type and similarity to preoperative seizures could not predict long-term seizure control (*p* = 0.93) 2. APOS onset < 24 h was significantly associated with a favorable seizure outcome as opposed to > 24 h (*p* = 0.013) 3. Occurrence of multiple postoperative seizures was more predictive of an unfavorable outcome compared to single seizures (*p* = 0.0035)
[10], Mayo Medical Center (USA)	Patients who had undergone temporal or frontal resection	Lesional pathology, mesial temporal sclerosis/gliosis, normal	Temporal lobectomy (33), frontal resection (12)	> 24	45	19	1. 5% of temporal lobectomy patients exhibited RDS vs. 15% of frontal resection patients (*p* = 0.009) 2. Preoperative IED in the resected temporal lobe improved postoperative seizure control (*p* = 0.03)
[6], Bethel Epilepsy Centre (Germany)	Adult patients who had undergone epilepsy surgery	N/A	Temporal lobectomy (36), extratemporal resection (24)	112 (± 35)	60	42	1. RDS and RUS showed a higher incidence in extratemporal compared to temporal lobe epilepsy; 25.7% vs. 19.5% and 10.6% vs. 8.5%, respectively 2. In patients following temporal resection bilateral interictal sharp waves were associated with RUS (*p* < 0.001) 3. Absence of GTCS was associated with RDS (*p* = 0.045) 4. History of febrile seizures was associated with RDS (*p* = 0.004) 5. History of head trauma was associated with RUS (*p* = 0.014)
[15], Bethel Epilepsy Centre (Germany)	Patients with medically intractable partial epilepsy who underwent localized resection aged > 14 years at surgery	Hippocampal sclerosis, focal cortical dysplasia, tumor, other etiologies unknown	N/A	60	28^d^	11^d^	The presence and absence of postoperative IED predicted RUS and RDS, respectively (*p* = 0.001)
[7], Bethel Epilepsy Centre (Germany)	Adult patients who underwent resective surgery for intractable partial epilepsy	Focal cortical dysplasia, tumor, gliosis, vascular malformation, Rasmussen syndrome, malformation of cortical development	Frontal lobe resective surgery, lesionectomy, cortical resection, lobectomy, lesionectomy with multiple subpial transections	83 (± 46)	44	6	1. Absence of IED after 6 months of follow-up was associated with RDS (*p* = 0.01) 2. No history of GTCS was associated with RDS (*p* = 0.015)
[33]Salanova et al., 1996, Montreal Neurological Institute (Canada)	Surgically treated patients with medically refractory temporal lobe epilepsy	N/A	Resection of temporal neocortex, amygdala, and/or most of hippocampus, extratemporal corticectomy	24–480	101	61	1. Bilateral IED were more frequent in patients with poor outcome (*p* < 0.001) 2. EZ size estimated through electrocorticography was positively associated with a worse prognosis 3. History of febrile seizures was associated with seizure freedom (*p* = 0.002) 4. History of head trauma or encephalitis was associated with persistent seizures (*p* = 0.010; *p* < 0.001) 5. Surgery at age < 30 years correlated with better seizure outcome when comparing seizure-free vs. not seizure-free patients (*p* = 0.007)
[17], Bethel Epilepsy Centre (Germany)	Temporal lobe epilepsy patients > 16 years who had seizures between 2–6 months postoperatively	Hippocampal sclerosis (46), CA4 sclerosis (6), tumor (16), vascular malformation (4), focal cortical dysplasia (5), posttraumatic lesions (4), polymicrogyria (1), ischemia (1), microdysgenesis (1), normal (2)	Temporal lobectomy (45), key-hole resection (18), lesionectomy (23)	24	86	27	Exclusively ipsilateral temporal IED were associated with seizure freedom (*p* = 0.009)
[35], Epilepsy Center Freiburg (Germany)	Patients with intractable mesiotemporal lobe epilepsy who underwent surgical treatment	Hippocampal sclerosis, focal cortical dysplasia, neoplastic lesion, vascular lesion	Amygdalohippocampectomy, lesionectomy, anterior temporal lobectomy, standard temporal lobectomy	> 60	54 (APOS defined as seizures < 1 month postoperatively)	33	Neoplastic lesions were associated with RDS (*p* = 0.08)
[22], Epilepsy Centre of the National Institute of Psychiatry and Neurology (Hungary)	Patients who underwent an anterior temporal lobectomy	Hippocampal sclerosis, cortical dysplasia, tumor, encephaloclastic lesion, cavernoma	Anterior temporal lobectomy (50)	73 (24–204)	50 (APOS defined as seizures < 1 month postoperatively)	3	RDS occurred exclusively in patients with hippocampal sclerosis
[38], Mayo Clinic (USA)	Patients who underwent frontal lobe cortical resection for intractable partial epilepsy	Tumor, vascular anomaly, malformation of cortical development, gliosis, normal	Frontal lobe cortical resection (17)	48 (12–144)	17	10	Timing of seizures in the first postoperative week had no prognostic value (*p* = 0.85)
[20], Cleveland Clinic Epilepsy Center (USA)	Patients who underwent resective epilepsy surgery who had at least one seizure > 7 days postoperatively	Malformation of cortical development, hippocampal sclerosis, tumor, vascular malformation, trauma, infection, normal	Lesionectomy, lobectomy, selective amygdalohippocampectomy, neocortical resection	82 (12–228)	276	95	1. Postoperative seizure onset < 6 months was predictive of an unfavorable outcome, excluding seizures within the first postoperative week (*p* < 0.001) 2. Having a second seizure < 6 months after surgery increased the risk of refractoriness (*p* < 0.001)
[4], Jefferson Comprehensive Epilepsy Center (USA)	Patients who failed to respond to > 1 anti-epileptic drug and had subsequent anterior temporal lobectomy	Normal, mesial temporal sclerosis, extratemporal abnormality	Anterior temporal lobectomy (136)	> 12	136	64	Longer latency to the first postoperative seizure was associated with seizure freedom (*p* < 0.001)
[23], University of California, Pediatric Epilepsy Surgery Program (USA)	Children who underwent hemispherectomy	Hemimegalencephaly, hemispheric cortical dysplasia, Rasmussen encephalitis, ischemia, other etiologies unknown	Hemispherectomy (26)	6–60	26	11	1. Patients with > 5 APOS had worse seizure control compared to those with 0–5 APOS (*p* < 0.0001) 2. Patients with > 5 APOS had a higher chance of reoperation compared to those with 0–5 APOS (*p* = 0.0094)
[8], Bethel Epilepsy Centre (Germany)	Adult patients > 16 years who underwent epilepsy surgery and displayed seizures < 6 months postoperatively	Hippocampal sclerosis (116), tumor (53), focal cortical dysplasia (47), vascular malformation (11), inflammation (8), gliosis (24), normal (4)	Frontal lobe resection (37), posterior cortical resection (34), multilobar resection (3), mesial resection (145), temporal lobe resection (47)	108 (± 36)	266	52	Preoperative tonic seizures were predictive of RUS (HR 0.14, 95% CI 0.03–0.052

^a^ *APOS* Acute postoperative seizures; *EZ* Epileptogenic zone; *GTCS* Generalized tonic–clonic seizures; *IED* Interictal epileptiform discharges; *RDS* Running-down seizures; *RUS* Running-up seizures

^b^ Patients presenting with APOS (onset < 7 days postoperatively), subacute (onset < 1 month postoperatively), or deferred seizures (onset < 6 months postoperatively). Studies analyzed either APOS or RDS following postoperative seizures with onset later than 7 days

^c^ A favorable seizure outcome was defined in studies as ILAE 1–3, Engel I, or Engel I-II[9, 40].

^d^ Patients with IED 6 months postoperatively, APOS were not specifically analyzed in this study

### APOS semiology

Postsurgical seizures present in a heterogeneous manner. Seizures have previously been categorized into either habitual, non-habitual, or neighborhood seizures, which all derive from different pathophysiological mechanisms [5]. Habitual seizures resemble non-habitual seizures. In the latter, however, epileptogenic discharges are propagated in an alternative manner due to surgical disturbances of the original pathways. This results in seizures different from presurgical semiology. Neighborhood seizures are precipitated by post-operative inflammation caused by e.g. surgical manipulation, hemorrhage, or infection of surrounding tissue. They present as focal simple motor seizures without loss of consciousness [5, 38]. Studies have been contradictory regarding the relevance of seizure semiology. Some conclude habitual seizures in the acute postoperative period decrease the odds of a favorable seizure outcome in comparison to non-habitual seizures, or when merely focal motor seizures and/or generalized tonic–clonic seizures (GTCS) occur [14, 21, 25]. Swanson et al. demonstrated a trend toward early generalized seizures predicting a more favorable outcome compared to focal seizures with impaired awareness [37]. However, these findings were not reproduced [11, 26, 27, 31]. Seizures provoked by low anti-epileptic drug levels, metabolic disturbances, or complications may be less likely to evolve into RUS [5]. Malla et al. found no significant difference in seizure outcome between patients with and without provoked seizures [25]. A later study by McIntosh et al. showed a trend toward a reduced risk of seizure recurrence in those with acute symptomatic seizures[27].

### Focus of epilepsy

Spatially defining the epileptogenic focus using EEG or MRI remains a key element in constructing a therapeutic strategy, but its merit for APOS is unclear. The incidence of RDS in temporal lobe epilepsy does not strongly differ from extratemporal lobe epilepsy; for either subtype most studies report RDS to occur in 5–26% of patients presenting with early postoperative seizures [3, 6, 10, 14, 19, 34, 36]. Ficker et al. found RDS were more likely to occur following frontal resection compared to temporal lobectomy [10]. This was reproduced by Elsharkawy et al., who demonstrated a higher incidence of both RDS and RUS in extratemporal compared to temporal lobe epilepsy [6]. Jeha et al. found that seizures are likely to recur earlier in patients with frontal as opposed to temporal lobe epilepsy [19].

#### EEG

Absence of interictal epileptiform discharges (IED) in the early postoperative period predicted RDS [7, 15]. Unilateral IED were more associated with a favorable outcome than bilateral IED in patients who experienced postoperative seizures, although these did not strictly concern APOS [6, 10, 17, 33]. Using intracranial EEG to demarcate the primary EZ, Salanova et al. demonstrated an association between RDS and an intermediately sized EZ, whereas patients with the smallest EZ were more likely to be completely seizure-free. Patients with persistent seizures had the largest EZ [33].

#### MRI

Schmeiser et al. showed patients with an underlying neoplasm were more likely to display RDS compared to those with non-neoplastic lesions such as hippocampal sclerosis, focal cortical dysplasia, or vascular lesions [35]. This contrasts with a study by Kelemen et al., in which the patients displaying RDS (*n* = 3) all had hippocampal sclerosis rather than a neoplastic lesion [22]. In some cases a morphological seizure correlate cannot be ascertained preoperatively. In these patients, the chances of refractoriness after early postoperative seizures are significantly higher [20, 22, 27].

### Timing of seizure recurrence

The definition of the APOS timeframe is debated. Although its most applied definition is the occurrence of seizures within one week postoperatively, as defined by Beghi et al., [2] studies sometimes employ broader thresholds. It is difficult to establish which is the most appropriate definition, as studies with variable thresholds show a similar unfavorable seizure outcome [12]. Few studies have shown any significant differences between early and late APOS in the context of seizure recurrence. Two studies with a pooled sample size of *n* = 61 showed patients displaying seizures < 24 h had a more favorable outcome compared to those with later seizures [11, 26]. This was not reproduced in four other studies with a pooled sample size of *n* = 155 [25, 27, 31, 38]. When applying the conventional APOS threshold, McIntosh et al. showed a trend in favor of seizures occurring > 7 days postoperatively compared to an onset < 7 days [27]. This result was not observed in a later study [14]. When applying a broader threshold, Jehi et al. found seizures occurring < 6 months postoperatively were predictive of an unfavorable outcome, but this analysis excluded seizures occurring in the first postoperative week [20]. Buckingham et al. found longer latency to the first postoperative seizure was associated with a more favorable prognosis [4].

### Seizure frequency

Garcia et al. demonstrated the occurrence of a single APOS was associated with a more favorable outcome compared to multiple events in adults [11]. Multiple seizures occurring < 6 months postoperatively were associated with a significantly higher rate of refractoriness compared to a single seizure [20]. In a pediatric population, Koh et al. did not observe a difference in seizure outcome between patients without APOS and those with 1–5 APOS. However, the occurrence of > 5 APOS was associated with worse long-term seizure management and a higher reoperation rate [23]. Other studies failed to find an association between APOS frequency and seizure recurrence [26, 27, 31].

### Patient characteristics

Elsharkawy et al. demonstrated a negative association between a history of GTCS and RDS in three separate studies [6–8]. Additionally, a history of febrile convulsions was associated with RDS following temporal lobe surgery, whereas a history of head trauma was associated with RUS [6]. Such was reproduced by Salanova et al., who additionally found prior encephalitis significantly increased the likelihood of an unfavorable seizure outcome, and resulted in a significantly worse prognosis compared to previous febrile seizures [33]. Swanson et al. reported age at seizure onset and a favorable course of disease following APOS were independently correlated, unlike sex or age at operation [37]. Salanova et al. reported that patients aged < 30 years at surgery had a higher chance of becoming seizure-free subsequent to initial seizures [33].

## Discussion

It has been well established that APOS predict an unfavorable long-term seizure outcome [12]. However, generalizing APOS may be inappropriate when establishing a prognosis, considering their heterogeneity in clinical presentation and disease course. As research on APOS subtypes is limited, our review demonstrates that an open question remains through which framework APOS should be classified to accurately predict their evolution to RDS/RUS.

The relationship between APOS semiology and RDS remains largely unexplored, as the few existing studies show conflicting results [11, 14, 21, 25–27, 31, 37]. This may be due to inadequate differentiation between neighborhood seizures and non-habitual seizures. Clinical studies are needed to establish whether non-habitual seizures/neighborhood seizures have a more favorable prognosis than habitual seizures. This would require an accurate framework to discriminate between these subcategories. Making the clinical distinction between neighborhood seizures and non-habitual seizures can be difficult, considering both types present differently compared to preoperative semiology. However, neighborhood seizures are typically focal simple motor seizures without loss of consciousness arising from tissue surrounding the original EZ [5, 38]. Additionally, the distinction can be made by investigating known triggers for neighborhood seizures, such as metabolic disturbances, hemorrhage, or infection [5]. Alternatively, EEG can be used to localize the epileptogenic focus, where overlap with the original EZ would further support the classification of the postoperative seizure as a neighborhood seizure.

EEG is used to localize an epileptogenic focus or objectify the spread of epileptogenicity. Presence and laterality of IED show considerable prognostic potential [6, 7, 10, 15, 17, 33]. The etiology of bilateral spiking is unclear, though it is hypothesized to originate from mirror foci caused by commissurally propagated discharges [29, 30]. Contralateral spiking can also derive from signal dissemination rather than extensive epileptogenesis, [16] though in this case the negative association between bilateral spiking and RDS suggests the latter. Salanova et al. illustrated the relevance of EZ size. In accordance with Rasmussen’s aforementioned theory they allocated the increase of EZ size to secondary epileptogenesis, reflecting a more advanced and intractable state of disease [33]. The measure to which these findings can be applied in the context of APOS is unclear. Prognostic studies on the variables mentioned above, along with other EEG-derived factors like resting-state activity, postictal changes, and seizure onset patterns are needed to substantiate the relevance of EEG in the acute postoperative period.

The feasibility of neuroimaging to predict seizure outcome is yet to be explored, though it can be argued from a theoretical perspective. Epilepsy-associated lesions show a vast heterogeneity in circumscription and their detrimental effect on adjacent tissue. Such differences in lesion characteristics would likely affect the susceptibility to postsurgical seizures. Future MRI studies will have to objectify whether lesion subtypes, lesion circumference, and postoperative presence of residual tissue are associated with RDS/RUS.

The timing of seizure recurrence does not seem relevant in predicting seizure outcome, though two studies demonstrated a negative association between late postoperative seizures and long-term seizure recurrence [4, 20]. It is worth noting the pathophysiological dissimilarities between early and late APOS have not yet been clarified. APOS frequency provides a promising alternative, as it has been numerously associated with seizure outcome using variable seizures frequency thresholds [11, 20, 23]. Considering the lack of consensus, a standardized threshold must first be established to facilitate using APOS frequency to predict RDS/RUS. APOS should be classified as postoperative seizures occurring < 7 days postoperatively, considering transient factors such as surgical trauma, inflammation, or edema are typically resolved by the end of this period. Seizures occurring between 7 days – 1 month postoperatively should be classified as “subacute”, reflecting those arising from delayed pathological changes, such as ischemia or neuroplastic alterations. Finally, seizures occurring between 1 – 6 months postoperatively should be classified as “deferred”, as these are more associated with chronic processes such as scar formation or the development of novel pathological structures.

Medical history was strongly correlated with seizure outcome. A history of either GTCS, head trauma, or encephalitis was associated with a more unfavorable outcome as opposed to prior febrile seizures, as these may indicate a more extensive spread of epileptogenicity [6–8, 33]. Retrospective studies are needed to identify other medical history components relevant to the progression to RDS/RUS. Age at seizure onset and age at surgery also showed a correlation with outcome. Younger age may indicate underlying pathology associated with a higher surgical success rate, such as focal cortical dysplasia, low-grade malignancies, or mesial temporal sclerosis [33, 37]. Janszky et al. investigated whether a change in anti-epileptic drug regimen influences the evolution to RDS, but a significant effect was not observed [17].

The prognostic value of the variables mentioned above has been demonstrated outside of the context of APOS or RDS. A meta-analysis by Tonini et al. showed MRI abnormalities, history of febrile seizures, EEG/MRI concordance, and presence of malignancy were associated with a favorable seizure outcome. The necessity of perioperative intracranial monitoring and postoperative epileptogenic discharges were more ominous predictors [39]. A meta-analysis on seizure outcome following repeated epilepsy surgery also showed congruent EEG, MRI abnormalities, and lesional pathology were associated with a favorable outcome. The use of invasive monitoring was associated with an unfavorable outcome, whereas the occurrence of generalized seizures merely trended towards seizure recurrence [24]. Seeing these variables are associated with seizure outcome in general would indicate they may not be directly implicated in the etiology of RDS, but rather exert an effect independently. Despite their unresolved etiological implications, studies indicate these variables are capable of predicting RDS/RUS and can nonetheless be utilized to distinguish favorable from unfavorable APOS.

Prior studies have demonstrated the feasibility of using a multifactorial model to predict postoperative seizures. Janszky et al. constructed a model including secondary GTCS preoperatively, seizures occurring < 6 months postoperatively, and ipsilateral temporal IED, resulting in an area under the curve of 0.78 [17]. A model by Jayalakshmi et al. integrated seizure presentation, IQ score, APOS, imaging abnormalities, and IED, reaching an area under the curve of 0.97 in a sample of pediatric patients [18]. Although these findings are promising, both models require testing in multiple, diverse populations before they can be considered valid. Prospective cohort studies are needed to assess the feasibility of applying predictive models integrating EEG-derived variables, APOS frequency, and medical history to predict seizure outcome.

### Limitations

The primary limitation of this review was that most of the included studies comprised a low proportion of patients exhibiting APOS. The resulting small sample of interest could explain some of the conflicting results. The measure to which several variables of interest were reported also greatly varied. A significant number of articles (*n* = 18) was excluded due to only their abstract being published, which in turn did not provide sufficient insight into study methodology and results. These factors have likely contributed to a low statistical power, underscoring the need for a meta-analysis which can provide a more precise estimate of the effect of variables of interest. Included studies were not graded on certainty of evidence, therefore no statements can be made on the measure of bias. Combined with differences in study methodology and population, the heterogeneity in APOS definition between studies does not permit an adequate pooling of results. All studies included were retrospective, disallowing any causal statements. Admittedly, given the limited incidence of APOS a prospective design would not be cost-effective. It would have been interesting to evaluate whether the association between seizure characteristics and clinical outcome is modified in a pediatric population. However, adult and pediatric study samples were generally composed of both patients with and without APOS, and individual patient data was not available. Additionally, included studies showed considerable heterogeneity regarding surgical procedure and pathological subtype. However, the role of surgical procedure, practice variations, or pathological subtype was not investigated in the included studies. Comparing these variables across studies was not feasible, as one-on-one comparisons between these small studies would not be valid. However, given the significant pathophysiological differences associated with these variables, understanding their role is crucial for accurately interpreting long-term seizure outcomes.

## Conclusion

The variability in clinical outcome following APOS delineates the necessity for a more accurate classification of postoperative seizures. Especially favorable prognostic factors could aid counseling in clinical practice, as surgical failure is not certain in APOS. Existing literature sheds light on numerous knowledge gaps, which offer valuable starting points for future research with the potential to advance our understanding of APOS and contribute meaningfully to this critical field (Table 3). Prior studies have shown EEG-derived variables (IED presence/laterality, EZ size), APOS frequency, and medical history (GTCS, head trauma, encephalitis) are associated with RDS/RUS as opposed to ictal expression and timing of seizure recurrence. However, the strength of these associations is uncertain due to small sample sizes, limited reporting of variables of interest, and strong between-study heterogeneity. Due to limited evidence, the relationship between MRI findings and seizure recurrence is still unclear. Future research should focus on constructing and validating a multifactorial model that integrates IED presence/laterality, EZ size, APOS frequency, and medical history components such as GTCS, head trauma, and encephalitis to more accurately predict long-term seizure outcome following resective epilepsy surgery.

**Table 3:** Summary of current knowledge gaps and starting points for future research

Knowledge gaps	Starting points for future research
The heterogeneity of APOS makes categorization into RDS/RUS challenging	Developing and validating a standardized classification system for APOS subtypes based on clinical and pathophysiological presentation
The relationship between APOS semiology and RDS	Prospective studies focused on establishing the relationship between habitual vs. non-habitual vs. neighborhood APOS and long-term seizure outcome
The prognostic value of EEG-derived variables (e.g. IED presence/laterality, EZ size, resting state activity, postictal changes, seizure onset patterns)	Clinical studies investigating the prognostic value of IED presence/laterality, EZ size, resting state activity, postictal changes, and seizure onset patterns for seizure outcome following APOS using a standardized protocol
The mechanism behind bilateral spiking and secondary epileptogenesis	Fundamental research focusing on bilateral spiking network dynamics, genetic/molecular pathways involved in secondary epileptogenesis, and the role of synaptic plasticity in the EZ and contralateral regions
The role of MRI in predicting seizure recurrence	MRI studies focused on investigating predictors (e.g. lesion subtypes, lesion circumference, and postoperative presence of residual tissue) for seizure recurrence in patients with lesional pathologies
Pathophysiological differences between early and late APOS	Clinical studies comparing the clinical presentation and/or physiological measures between early vs. late APOS to determine prognostic implications
A prognostic threshold for APOS frequency or timing of onset does not exist	Clinical studies focused on identifying clinically relevant cut-offs for APOS frequency and timing of APOS onset through subgroup analyses
The impact of practice variations, pathology subtype, and surgical procedure on APOS outcome	Prospective studies focused on the influence of practice variations, pathology subtype, and surgical procedure on seizure outcome after APOS
The implication of medical history components in APOS evolution to RDS/RUS	Retrospective studies focused on clarifying which medical history components are associated with seizure outcome after APOS
The feasibility of combining EEG-derived variables, APOS frequency, and medical history to predict long-term outcome	Applying multivariate models in prospective cohorts which integrate EEG-derived variables, APOS frequency, and medical history for outcome prediction
	Prognostic studies investigating the use of machine learning to integrate imaging, EEG, and clinical data to predict evolution to RDS/RUS

^a^ *APOS* Acute postoperative seizures; *EZ* Epileptogenic zone; *IED* Interictal epileptiform discharges; *RDS* Running-down seizures; *RUS* Running-up seizures

## Supplementary Information

Below is the link to the electronic supplementary material.

ESM 1 (DOCX 14.9 KB)

## Data Availability

No datasets were generated or analysed during the current study.

## Abbreviations

APOS: Acute postoperative seizures
EZ: Epileptogenic zone
GTCS: Generalized tonic–clonic seizures
IED: Interictal epileptiform discharges
RDS: Running-down seizures
RUS: Running-up seizures
