# Supplementary material for: Do acute postoperative seizures predict epilepsy surgery outcome? a scoping review
Source: Acta Neurochir (Wien). 2025 Mar 13;167(1):72. doi: 10.1007/s00701-025-06486-8 (PMC11903598; doi:10.1007/s00701-025-06486-8)
Supplement: Supplementary file 1 — (DOCX 14.9 KB) [file 701_2025_6486_MOESM1_ESM.docx]

Article title: Do acute postoperative seizures predict epilepsy surgery outcome? A scoping review.

Journal name: Acta Neurochirurgica

Author names: Sebastiaan E. A. van Maanen, Maeike J. M. Zijlmans, Pieter van Eijsden, Sandra M. A. van der Salm

Affiliation corresponding author: Department of Neurology and Neurosurgery, Utrecht Brain Center, University Medical Center Utrecht, Utrecht, Netherlands

E-mail address corresponding author: s.e.a.vanmaanen@umcutrecht.nl

**Online Resource, Methods**. The supplementary information expands on the Methods provided, as it provides the search strings applied in the PubMed & Embase databases.

**Search strings PubMed & Embase**

*PubMed*

(Epilep*[tiab] OR "Seizure Disorder*"[tiab] OR Aura*[tiab] OR seizure*[tiab] OR convuls* [tiab) AND (((("focal"[tiab] OR temporal[tiab] OR frontal[tiab] OR parietal[tiab] OR occipital[tiab]) AND resection*[tiab]) OR topectom*[tiab] OR lobectom*[tiab] OR transection*[tiab] OR amygdalohippocampectom*[tiab] OR lesionectom*[tiab]) OR (surg*[tiab] n10 epilepsy[tiab])) AND ("running down phenomenon"[tiab] OR "running-down phenomenon"[tiab] OR ((“running-down seizure*”[tiab] OR “running down seizure*”[tiab]) OR ((“post-operative seizure*”[tiab] OR “postoperative seizure*”[tiab] OR “post operative seizure*”[tiab] OR “post-resection seizure*”[tiab] OR “postresection seizure*”[tiab] OR “post resection seizure*”[tiab]))))

*Embase*

(Epilep*:ab,ti OR "Seizure Disorder*":ab,ti OR Aura*:ab,ti OR seizure*:ab,ti OR convuls*:ab,ti) AND ((((focal:ab,ti OR temporal:ab,ti OR frontal:ab,ti OR parietal:ab,ti OR occipital:ab,ti) AND resection*:ab,ti) OR topectom*:ab,ti OR lobectomy*:ab,ti OR transection*:ab,ti OR lesionectom*:ab,ti OR amygdalohippocampectom*:ab,ti) OR ((surg* NEAR/10 epileps*):ab,ti)) AND ("running down phenomenon":ab,ti OR "running-down phenomenon":ab,ti OR ((“running-down seizure*”:ab,ti OR “running down seizure*”:ab,ti) OR ((“post-operative seizure*”:ab,ti OR “postoperative seizure*”:ab,ti OR “post operative seizure*”:ab,ti OR “post-resection seizure*”:ab,ti OR “postresection seizure*”:ab,ti OR “post resection seizure*”:ab,ti))))
